# A Case Report of Central Nervous System Graft-Versus-Host Disease and Literature Review

**DOI:** 10.3389/fneur.2021.621392

**Published:** 2021-03-10

**Authors:** Mingming Li, Yue Zhang, Yujia Guan, Zunwei Zhang, Hanbing Dong, Yang Zhao, Hui Deng

**Affiliations:** Department of Neurology, First Affiliated Hospital of Jilin University, Changchun, China

**Keywords:** graft-versus-host disease, central nervous system, diffuse white matter lesions, immunosuppression, bone marrow transplantation

## Abstract

As an adverse immune phenomenon, graft-versus-host disease often occurs after allogeneic hematopoietic stem cell transplantation. The incidence of acute and chronic graft-versus-host disease is about 40–60% and the mortality rate can reach 15%, which is a potentially fatal disease. There are rare GvHD cases involving the central nervous system. We reported a rare case of diffuse white matter changes after haploid bone marrow transplantation, summarizing its clinical manifestations and diagnosis and treatment in conjunction with the literature.

## Introduction

A 22-year-old woman suffered headaches, vomiting, progressive unconsciousness, left hemiplegia and dysarthria. She was diagnosed as leukemia and received an allogeneic hematopoietic cell transplantation (HCT). Neurological examination showed drowsiness, dysphoria, dysphasia, gaze to the right, left hemiplegia, positive left Babinski sign, and positive Kernig sign. Brain magnetic resonance imaging (MRI) showed diffuse white matter and corpus abnormal signal. Intracranial pressure was more than 400 mmH_2_O. Cerebrospinal fluid (CSF) test revealed normal protein and white blood cell, mildly increased glucose, and elevated immunoglobulin G.

### Case Presentation

A 22-year-old woman was accepted to our department for the main complaint of paroxysmal occipital headache and vomiting, following progressive unconsciousness, left hemiplegia and dysarthria for 5 days. For medical history, she was diagnosed as acute myeloid leukemia (AML) M5 3 years ago and received induction remission treatment 1 week after the diagnosis. After that, she underwent intermittent chemotherapy for 10 months (until 2 years and 1 month before admitted) to achieve complete remission and then received a haploid hematopoietic stem cell transplant with father donor. Liver acute GVHD occurred in the 4th week after transplantation (2 years before admitted), and lung injury manifested as bronchiolitis obliterans in the 5th month (1 year and 8 months before admitted). Chronic GvHD involving the gastrointestinal tract and skin appeared at 1 year and 6 months before admitted. In the following 6 months, the symptoms of GVHD were relieved after treatment with corticosteroids and cyclosporine as anti-rejection drugs. Regular examination of bone marrow puncture showed a state of remission. Anorexia symptoms appeared when steroids gradually decreased, and central nervous system symptoms appeared 1 month later. Neurological examination showed drowsiness, dysphoria, dysphasia, gaze to the right, left hemiplegia, positive left Babinski sign, and positive Kernig sign.

**Figure 1:**
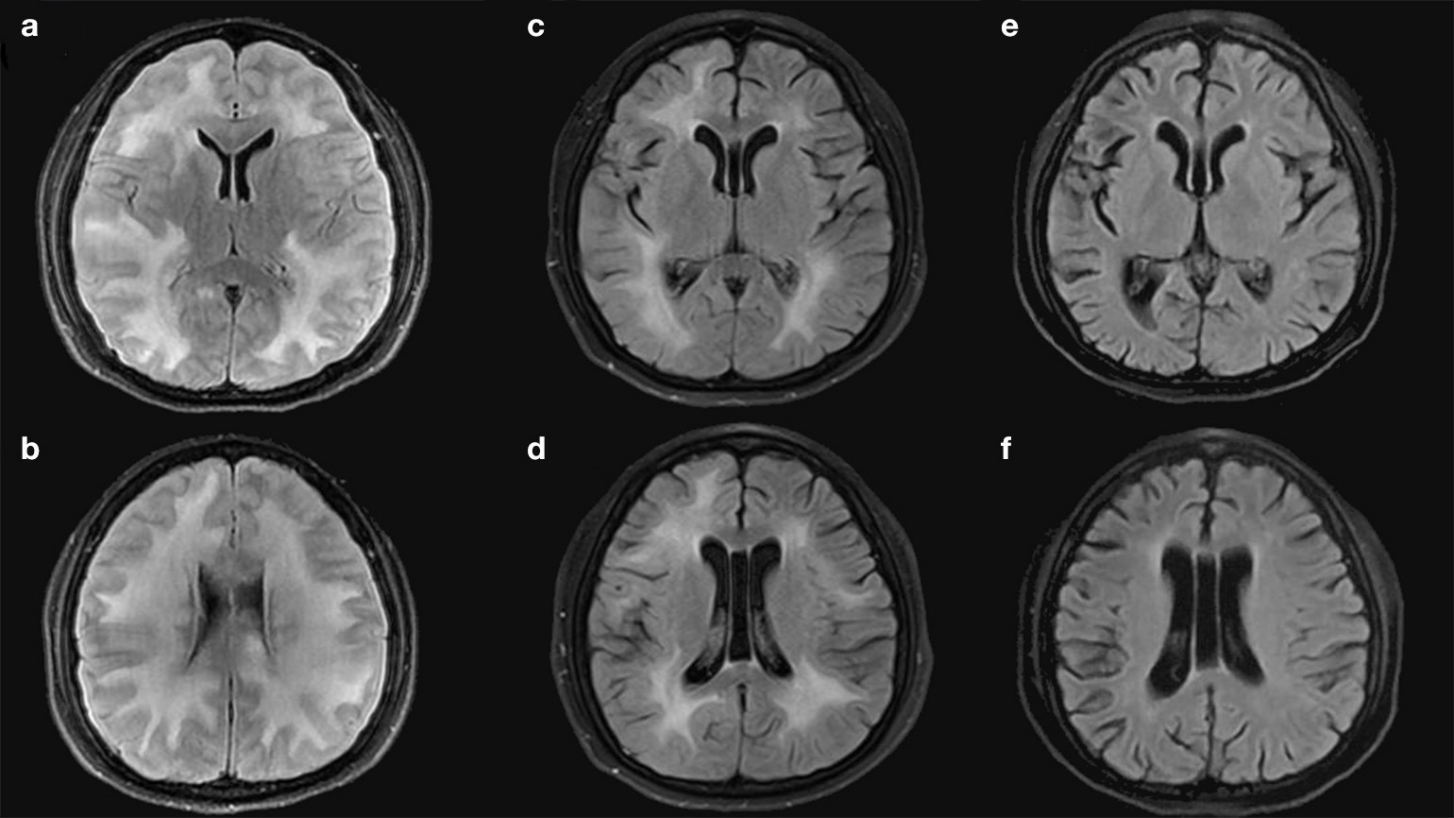
**(a–f)** MRI done in different periods.

Brain magnetic resonance imaging (MRI) showed diffuse white matter and corpus abnormal signal with slightly lower T1WI signal, slightly higher T2WI and FLAIR signal, bilateral lateral ventricles and partial sulcus were narrowed, and cerebral gyrus was swelling (Figures 1a,b). The dark-fluid presents a roughly symmetrical high signal, the ADC signal is slightly higher, and DWI has no dispersion limitation. Laboratory examination including rheumatoid factor, antinuclear antibody, antineutrophil cytoplasmic antibody, single and double strand DNA revealed almost normal exclude mild hyponatremia (Na^+^ 131.2 mmol/L, normal range 137–147 mmol/L). Serum cryptococcal antigen was not detected and the panel PCR assay (including IgM Antibody assay for AdV, HSV, RuV, RSV, toxoplasma, Legionella, and PCR for EBV, CMV, VZV, HHV) revealed negative. Intracranial pressure was more than 400 mmH_2_O. Cerebrospinal fluid (CSF) test revealed normal protein (0.40 g/L) and white blood cell (WBC, 4 × 106 /L), mildly increased glucose (4.6 mmol/L, normal range 2.3~4.1 mmol/L), and elevated immunoglobulin G (IgG, 61.3 mg/L, normal range 0–34.0 mg/L). No malignant phenotype was found in flow cytometry. Autoimmune disease markers of both serum and CSF were also negative, which including NMDA-R-Ab, CASPR2-Ab, AMPA1-R-Ab, AMPA2-R-Ab, LGl1-Ab, GABAB-R-Ab, and GAD 65-Ab. Besides, serum toxicological screening was also negative. The patient was diagnosed with white matter lesions, and we speculate that it is more likely to be caused by central nervous system graft-versus-host disease.

**Figure 2:**
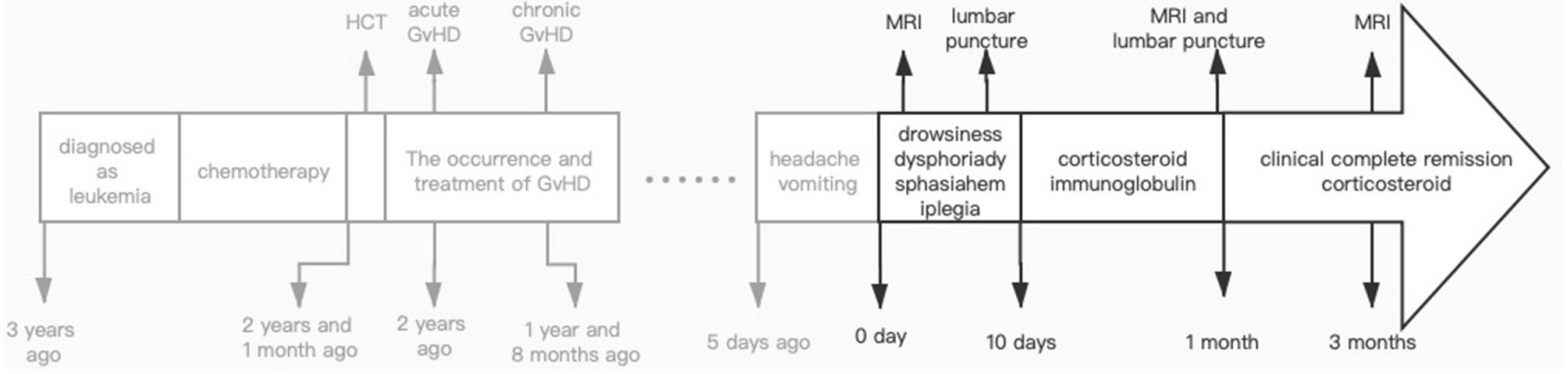
Historical and current information from this episode of care organized as timeline.

In addition to the ongoing systemic immunosuppression, the patient received intravenous corticosteroid (methylprednisolone 80 mg/day) for 14 days, followed intravenous immunoglobulin (0.4 g per kilogram of body weight) for 5 days. Consciousness and hemiplegia improved significantly within 2 days. After 3 weeks of treatment, the paralysis symptom was completely recovered, and anorexia and vomiting were relieved. Brain magnetic resonance imaging (MRI) appearances were slightly improved, edema lessened than before (Figures 1c,d). Reexamination of lumbar puncture showed that the cranial pressure was lower (250 mmH_2_O). Cerebrospinal fluid test showed decrease IgG (44.4 mg/L). Intravenous hormone dosage decreased to methylprednisolone 40 mg/day and sequentially reduced. A follow-up brain MRI, at 3 months from the start of methylprednisolone treatment (Figure 2), showed disappearance of lesions in the brain white matter (Figures 1e,f).

### Short Review of GvHD

As a rather common treatment of blood system diseases nowadays, allogeneic HCT still has its limitation. Its main obstacle is graft-versus-host disease (GvHD), an immune-mediated disease that could affect many tissues and organs (1). Among patients receiving HCT, the incidence of GVHD is as high as 40 to 60% (2, 3). In this potentially fatal disease, the mortality rate may be close to 15% (2). Studies also confirmed the importance of donor selection. The prevalence of acute GVHD and chronic GVHD and treatment-related mortality increased significantly in recipients of HLA mismatched donors (4). Although the neurological complications of acute and chronic graft-versus-host disease are rare, they can significantly affect the quality of life and have a high mortality rate (5).

The diagnostic criteria for acute CNS-GvHD disease have not been clearly proposed in the literature. Openshaw described six diagnostic criteria for chronic CNS-GVHD in 2009, including: 1. Occurrence with chronic GVHD affecting other organs; 2. Neurological signs of CNS involvement without other explanation; 3. Corresponding brain MRI abnormality; 4. Abnormal CSF studies (pleocytosis, elevated protein or immunoglobulin G, oligoclonal bands); 5. Pathological brain biopsy or post-mortem examination; 6. Response to immunosuppressive therapy (6). In 2010, a report from the Consensus Conference on Clinical Practice in chronic CNS-GvHD suggested that for clinical purposes, chronic CNS-GVHD can be diagnosed by meeting two of the first two criteria and at least two of the four remaining criteria (5).

Relevant articles were selected by searching the keyword “central nervous system GvHD” for relevant case reports via pubmed. Then we reviewed references of all selected articles to research additional articles. We selected 29 articles published between 1990 and December 2019, including 46 cases (Table 1) (7–35), 22 with histological analysis (brain biopsy, spine biopsy, or autopsy) (7, 8, 10, 13, 17–19, 22, 25, 27, 29, 30, 32–34). Patients who had received an allogeneic HCT for hematological disease were selected if they had CNS abnormalities and other diagnosis (autoimmune disease, infectious diseases, hematologic malignancies relapse, or post transplantation lymphoproliferative disorders) were excluded. In these cases, the male: female ratio was 1.42, which shows that males account for more. The median age of onset was 41 years old (range 9~68 years old). Primary diseases mainly include hematological malignancies and a few other diseases that require stem cell transplantation (Supplementary Chart 1). Sixteen patients received bone marrow from matched-related donor, 11 patients received from matched-unrelated donor, four patients received from mismatch-unrelated donor, two patients received cord blood, one patient received one-allele mismatched related donor, and one patient received haplo-identical T depleted cells. A total of 25 cases reported the history of acute GvHD. In addition, 29 cases reported the onset of chronic GvHD before or during the onset of neurological symptoms. In these cases, median neurology symptoms onset was 390 days after HCT (7-7300). In order to prevent or treat immune rejection, immunosuppression therapy is widely used in patients undergoing bone marrow transplantation. It is also considered to be one of the trigger factors of graft-versus-host disease (36). In these cases, 40 patients received immunosuppressive therapy to prevent GvHD, and 16 of them stopped immunosuppressive therapy before the onset of the disease. The main clinical manifestations vary, we made statistics on the patients' neurological symptoms (Supplementary Chart 2). Their clinical characteristics are heterogeneous: 11 of these showed a stroke-like episodes or lacunar syndrome, 13 patients had ADEM or multiple sclerosis-like presentation, 17 patients had symptoms of encephalopathy or encephalitis, and the clinical manifestations of the remaining four patients were not typical. Twelve patients underwent electroencephalograms (EEG). Ten people's EEG showed diffuse slow wave activity, and two of those ten detected seizures. The other two EEG results showed that one was conformed encephalitis, and the other has no obvious abnormality. A total of 40 patients were tested for cerebrospinal fluid. Eleven of them (27.5%) had no abnormalities, and elevated protein (*n* = 23, 57.5%) was the most common abnormality (Table 2). Except for the first patient reported in 1990, the remaining 45 people all underwent brain MRI, only three people indicated normal. Abnormal imaging manifestations are mainly divided into three categories, hemorrhage or ischemia, white matter lesions, and others (Supplementary Table 1). The features of brain biopsy or autopsy data included: Perivascular inflammation (*n* = 16, 72.8%), vasculitis (*n* = 4, 18.2%), gliosis, microglia proliferation or activation (*n* = 8, 36.4%), infiltration of CD3^+^ / CD4^+^ T cells (*n* = 1, 4.5%), infiltration of CD3^+^ /CD8^+^ T cells (*n* = 6, 27.3%), parenchyma lymphocytic infiltration (*n* = 4, 18.2%), demyelination (*n* = 7, 31.8%), granulomatous infiltration (*n* = 3, 13.6%). Among the available information, 41 cases reported treatment methods and 40 of them received immunosuppressive treatment (Supplementary Table 2). We have ascertained the treatment effects and prognosis of all cases, three of them are not available. Most patients have achieved complete response (*n* = 15) or partial response (*n* = 17) in clinical and/or imaging studies after treatment, while other patients showed stable (*n* = 3), progress (*n* = 6), or transient remission (*n* = 2), respectively. Since only part of cases have been followed up, and the follow-up time ranges from 3 months to 6 years, it is difficult for us to conduct an effective prognosis assessment. At the end of the follow-up, six patients died and one patient survived (Supplementary Table 3).

**Table 1:** Detailed information of the cases.

**Time of report**	**Age**	**Sex**	**Initial disease**	**GvHD history**	**Clinical characteristics, imaging, and biological abnormalities**	**Histology**	**Immunosuppressive therapy**	**Outcome**
1990 (7)	32	m	CML	Chronic GvHD	Clinic: dysphagia, dysarthria; CSF: normal	Perivascular infiltrates of CNS	NA	Deceased
1993 (8)	9	f	Posthepatitis aplastic anemia	Chronic GvHD	Onset: 8 mo after HSCT; Clinic: seizure, spasticity, disturbance of consciousness; CSF: pleocytosis, elevated protein; MRI: cortical atrophy, ventricular dilatation	Diffuse infiltration of white matter with CD3 lymphocytes, Inflammatory cell perivascular infiltration, Focal CD68+ microglia proliferation (HLA-DR+)	NA	Deceased 13 mo after neurological symptoms of liver and kidney failure
1993 (8)	13	m	ALL	Chronic GvHD	Onset: 100 d after HSCT; Clinic: cognitive deficits; MRI: white matter lesions in cerebellum	Diffuse CD3 lymphocytes infiltration and gliosis, inflammatory cells were mostly confined to perivascular spaces	NA	Deceased 5 mo after neurological symptoms of interstitial pneumonia
1996 (9)	14	f	Lymphoblastic lymphoma B	Acute GvHD	Onset: 71 d after HSCT; Clinic: disorientation and myoclonus; CSF: elevated protein (0.9 g/L), elevated CSF Ig; MRI: multiple foci of hyperintense signal on T2-weighted images within the brain stem and deep white matter; EEG: diffuse slowing	No	MP 1g/d	Clinical and MRI PR Deceased 123 d after neurological symptoms of severe sepsis
1999 (10)	43	m	CML	Acute and chronic GvHD	Onset: 18 mo after HSCT Clinic: acute vertigo, hemiparesis, aphasia, cortical blindness; MRI: multiple hematoma; CSF: elevated protein Angiography: normal	T cells, B cells, and monohistiocytes vessel wall and perivascular infiltration, sporadic demyelination and micro glia reaction	Corticosteroids (1.5mg/kg) and cyclophosphamide (bolus)	CR Deceased 5 mo after neurological symptoms of pneumonia
1999 (10)	32	f	AML	Acute and chronic GvHD	Onset: 28 mo after HSCT; Clinic: aphasia, hemiparesis and seizure; MRI: ischemic areas, white matter lesion; CSF: normal	No	Corticosteroids (1.5mg/kg) and cyclophosphamide (bolus)	Progression with cerebral infarctions Deceased of tentorial herniation
1999 (10)	19	m	ALL	Chronic GvHD	Onset: 31 mo after HSCT; Clinic: sub-acute confusion, spastic right hemiparesis Perfusion; MRI: leukoencephalopathy; CSF: normal; Angiography: normal	No	Corticosteroids (1.5mg/kg)	CR
1999 (10)	32	m	CLL	Acute and chronic GvHD	Onset: 5 y after HSCT; Clinic: aphasia, apraxia, dementia, tetra paresis; MRI: leukoencephalopathy, hemorrhage; CSF: normal Perfusion; Angiography: normal	No	Corticosteroids (1.5mg/kg) and cyclophosphamide (bolus)	Clinical and MRI stability
1999 (10)	53	m	CLL	Acute and chronic GvHD	Onset: 30 mo after HSCT; Clinic: acute aphasia and cognitive deficit; MRI: periventricular white matter lesion, frontoparietal ischemia; CSF: pleocytosis; Angiography: MCA branch occlusion	No	NA	Progression
2000 (11)	22	f	ALL	Acute GvHD	Onset: 9 mo after HSCT; Clinic: hemiparesis MRI: white matter lesions in frontal lobe Angiography: multiple stenosis and occlusions in the peripheral branches of the anterior and middle cerebral arteries	No	Corticosteroids	Transient improvement
2001 (12)	25	m	T-cells lymphoma	Chronic GvHD	Onset: 380 d after HSCT Clinic: cerebellar and pyramidal syndromes and peripheral neuropathy CSF: oligoclonal bands, pleocytosis MRI: multiple	No	MP 1g/d and plasmapheresis	Clinical PR and MRI stability
					demyelinating-like, asymmetric, hyperintense areas on T2-weighted images at the brainstem, cerebellar and corona radiata white matter.			
2002 (13)	18	f	AML	Acute GvHD	Onset: 2 mo after HSCT Clinic: seizure, spastic, cognitive dysfunction MRI: diffuse atrophy EEG: bilateral slowing and voltage suppression over the left hemisphere CSF: elevated protein and pleocytosis	Cerebral and meningeal angiitis, granulomatous infiltration, lymphocytic infiltration with microglial proliferation	Corticosteroids	PR
2003 (14)	51	f	AML	Acute and chronic GvHD	Onset: 10 mo after HSCT Clinic: diplopia, dysarthria, and gait disturbance MRI: multifocal abnormal high signal intensity mainly in the white matter of both cerebral hemispheres as well as in the cerebellum and brainstem CSF: normal	No	NA	Clinical CR and MR PR Alive 15 mo after
2005 (15)	55	f	NHL	Chronic GvHD	Onset: 23 mo after HSCT Clinic: acute cerebellar syndrome CT: acute intra_x005f parenchymal hemorrhage in left cerebellum MRI: white matter lesions peri-ventricular Angiography: aneurysm of the left posterior inferior cerebellar artery and dilated branches of cerebral arteries	No	Tacrolimus	PR
2006 (16)	48	m	NHL	Acute GvHD	Onset: 14 mo after HSCT Clinic: headaches, personality change, cognitive dysfunction MRI: diffuse deep white matter changes with periventricular predominance CSF: pleocytosis, elevated protein EEG: slow wave activity consistent with a diffuse encephalopathy	No	MP 2 mg/kg	CR
2007 (17)	58	f	ALL	Acute and chronic GvHD	Onset: 178 d after HSCT Clinic: encephalopathy and seizure CSF: elevated protein (0.67 g/L) MRI: patchy areas of white matter lesions	Leptomeningeal perivascular infiltration of CD3+/CD8+ T cells	Corticosteroids	Transient improvement Deceased 123 d after neurological symptoms of multiorgan failure
2007 (17)	45	f	T-cells lymphoma	Acute and chronic GvHD	Onset: 18 mo after HSCT; Clinic: right hemiparesis and seizure; CSF: normal; MRI: white matter lesions in fronto-parietal lobe	Perivascular inflammation mainly composed of CD3+/CD4+ T cells of the donor (FISH XY)	MP 1g/d(5d)	Clinical then MRI CR Alive 8y after HSCT
2007 (18)	41	m	Follicular lymphoma	Chronic GvHD	Onset: 3 year after; HSCT Clinic: progressive left hemiparesis; CSF: normal; MRI: large mass of right parietal lobe	Focal infiltration of lympho_x005f histiocytic inflammatory cell and noncaseating granuloma with perivascular predominance IHC: CD3+ T cells	Corticosteroids and ciclosporin	Clinical and MRI PR
2009 (19)	56	m	NHL	Chronic GvHD	Clinic: dizziness, tinnitus, vertigo, proximal weakness; MRI: large lesion of the corpus callosum	Perivascular inflammation and scattered CD3+/CD8+ T cells associated with microglia activation and macrophages in brain parenchyma	Corticosteroids and MMF	Progression Deceased 2 mo later
2009 (20)	32	f	MDS	Chronic GvHD	Onset: 7 mo after HSCT; Clinic: bilateral papillar edema with almost blindness, weakness of lower limbs, urinary retention; CSF: normal; MRI: multiple white matter lesions mainly in internal capsule, thalamus and thorax spine, evocative of multiple sclerosis	No	Corticosteroids (bolus then 0.5 mg/kg) and ciclosporin	Clinical and MRI PR Alive 2y after HSCT
2009 (21)	40	f	Follicular lymphoma	Acute GvHD	Onset: 10 d after HSCT; Clinic: encephalitis and seizure; CSF: elevated protein (6.75 g/L), pleocytosis (96.8% of donor cells); MRI: normal	No	Corticosteroids (3 bolus) followed by etoposide (50 mg/m2) because of HLH evidence	Progression Deceased 32 d after HSCT
2010 (22)	35	m	CML	Acute and chronic GvHD	Onset: 4 y after HSCT; Clinic: seizure; MRI: cortical/sub cortical acute ischemic lesions in peri-insular region, left frontal and parietal lobe; EEG: temporal slowing without epileptic discharges	Micro-angiopathy	Corticosteroids (3 bolus then 1mg/kg) and cyclophosphamide (bolus) Then methotrexate (10mg/week)	CR
2010 (22)	28	f	AML	Acute and chronic GvHD	Onset: 2 y after HSCT; Clinic: progressive depression, cognitive deficits, cortical blindness, seizure, ataxia, tetraparesis; CSF: elevated protein, oligo-clonal bands; MRI: leukoencephalopathy then internal brain atrophy; EEG: generalized slowing and epileptic discharges	Perivascular infiltration of donor CD3+ CD8+ lympho-monuclear cells (FISH XY), loss of myelin and axon preservation	Corticosteroids (5 bolus then 1mg/kg) and cyclophosphamide (1 bolus then 100mg/j)	CR
2010 (22)	20	m	SCID	Chronic GvHD	Onset: 20 y after HSCT; Clinic: hemiparesis, ataxia, cortical blindness and deafness; CSF: elevated protein, pleocytosis; MRI: multiple focal ischemic lesions and hemorrhage	Perivascular infiltration of CD3+ CD8+ lympho-monuclear cells, loss of myelin and axon preservation	Corticosteroids and cyclophosphamide (4 bolus)	PR Deceased 1 y after neurological symptoms of severe sepsis
2010 (22)	33	m	CLL	Acute and chronic GvHD	Onset: 2 y after HSCT; Clinic: ataxia, cortical blindness, spastic tetraparesis, acute pseudo-bulbar syndrome; CSF: elevated protein, pleocytosis, intrathecal IgG synthesis; MRI: frontally accentuated brain atrophy	Cerebral and meningeal angiitis	Corticosteroids (3 bolus then 1.5mg/kg) and cyclophosphamide (1 bolus)	Small improvement Deceased 4 y after neurological symptoms of acute aspiration
2010 (23)	57	m	CMML	No	Onset: 4 w after HSCT Clinic: recurrent myelitis with mild paraparesis, urinary difficulty; CSF: elevated protein; MRI: multiple white matter lesion of spinal cord without cerebral anomalies	No	Corticosteroids (5 bolus then 1mg/kg) then cyclophosphamide (7 bolus)	CR but relapse of myelitis 1,5 y later Treatment by cyclophosphamide with PR
2010 (23)	65	m	AML	No	Onset: 3 y after HSCT Clinic: recurrent myelitis with mild paraparesis and lower limb hypoesthesia; CSF: elevated protein and oligoclonal bands; MRI: multiple white matter lesion of spinal cord, T2-hyperintense paraventricular left hemispheric lesion	No	Corticosteroids (5 bolus)	CR but relapse of myelitis 1 mo after Treatment by corticosteroids with CR
2012 (24)	54	m	AML	No	Onset: 390 d after HSCT; Clinic: progressive ascending weakness, areflexia and cranial neuropathy; CSF: elevated protein; MRI: multiple	Loss of myelin and axon preservation	Corticosteroids and IV Ig (5 courses)	PR Deceased 5 y later
					sub-cortical lesions, one with a relatively open ring sign			
2012 (24)	59	m	AML	No	Onset: 240 d after HSCT; Clinic: progressive ascending weakness, areflexia and cranial neuropathy; CSF: elevated protein, oligoclonal bands; MRI: pontine white matter lesion	No	IV Ig (5 courses)	PR Alive 9 y later
2012 (24)	29	m	AML	No	Onset: 63 d after HSCT; Clinic: progressive ascending weakness, areflexia and cranial neuropathy; CSF: elevated protein, oligoclonal bands; MRI: multiple white matter lesions of cervical spine and few in brain	Biopsy of spinal cord Loss of myelin and axon preservation	Corticosteroids and IV Ig (5 courses)	PR Deceased 2 y later of severe chronic GvHD
2014 (25)	63	m	CLL	Acute GvHD	Onset: 92 d after HSCT; Clinic: cognitive impairment, tremor; CSF: elevated protein; MRI: multifocal subcortical and juxtacortical white matter lesions	No	Prednisolone 0.5 mg/kg/d	CR Alive 1 y after
2015 (26)	7	m	Idiopathic aplastic anemia	Chronic GvHD	Onset: 15 mo after HSCT; Clinic: depression and seizure; CSF: normal; MRI: bilateral uncus lesions; EEG: slowing background and one epileptic focus VGKC and ILGI1 antibodies positives	No	Corticosteroids (5 boluses then 1mg/kg) and IV Ig (5 courses)	Clinical PR
2017 (27)	33	m	Fanconi disease	Acute and chronic GvHD	Onset: 308 d after HSCT; Clinic: myelitis with motor and sensitive neuronopathy, memory disorders; MRI: Pan-myelitis and posterior lepto-meningitis; EMG: Motor and sensitive neuronopathy and muscular junction damage; CSF: Pleocytosis with majority of T cells, elevated protein, oligoclonal bands	Lymphohistiocytic vasculitis without necrosis, perivascular infiltration with CD3+ CD8+ T cells	Corticosteroids (10 bolus then 1mg/kg), IV Ig (3 courses), plasmapheresis (10 courses), MMF	Clinical stability Alive 37 m after CNS symptoms
2017 (27)	62	m	MPN	Acute GvHD	Onset: 152 d after HSCT; Clinic: confusion, coma; MRI: Normal; EEG: diffuse brain suffering and frontal peak-waves discharges; CSF: Pleocytosis and elevated protein	Diffuse lymphocyte T infiltrate with small perivascular predominance and diffuse gliosis	Corticosteroids (1mg/kg)	Progression Deceased 17 d after CNS symptoms
2017 (27)	68	f	MPN	Acute GvHD	Onset: 9 d after HSCT; Clinic: encephalitis; MRI: Hyper T2 focal lesion of the left hemisphere; EEG: Encephalitis; CSF: Pleocytosis	No	Corticosteroids (2mg/kg)	Progression Deceased 5 d after CNS symptoms
2017 (27)	29	f	Fanconi disease	Chronic GvHD	Onset: 378 d after HSCT; Clinic: cerebellar syndrome, cranial nerves deficits and atypical poly-radiculonevritis; MRI: normal; CSF: elevated protein	No	Corticosteroids (3 bolus then 1mg/kg), plasmapheresis (5 courses), IV Ig (6 courses)	PR Deceased 30 mo after CNS symptoms
2017 (27)	50	m	MPN	Chronic GvHD	Onset: 2090 d after HSCT; Clinic: transient and focal deficits (right hemiparesis and paresthesia); MRI: T2 hyper-signals compatible with multiple sclerosis; CSF: normal	No	Ciclosporin A (6mg/kg)	CR Alive 8, 3 mo after CNS symptoms
2017 (27)	16	f	AML	Acute GvHD	Onset: 255 d after HSCT; Clinic: encephalitis, extra pyramidal syndrome; MRI: peri-ventricular and posterior leuko-encephalopathy associated with hemispheric cerebellar lesions with contrast enhancement; EEG: Global and diffuse slowing	No	Corticosteroid (1mg/kg)	PR Deceased 4, 2 mo after CNS symptoms
2017 (27)	36	m	CML	Acute and chronic GvHD	Onset: 199 d after HSCT; Clinic: cerebellar and vestibular syndromes, left hemiparesis and hypoesthesia, and cranial nerves deficits; MRI: lacunar infarct, cerebral vasculopathy associated with focal lesion Angiography: normal; CSF: Elevated protein with IgG polyclonal	No	Corticosteroids (1mg/kg)	PR then secondary aggravation Deceased 8 mo after CNS symptoms
2017 (28)	66	m	AML	Acute GvHD	Onset: 10 mo after HSCT; Clinic: drowsiness, apathy, diffuse cognitive impairment, frontal lobe involvement; MRI: hyperintensities of the centrum ovule and the lateral ventricles without gadolinium enhancement; CSF: normal; EEG: unremarkable	No	Corticosteroids (1mg/kg),fingolimod (0.5 mg/d)	CR but relapse of myelitis 7 mo later Alive 2, 3 y after CNS symptoms
2017 (29)	46	f	AML	Acute and chronic GvHD	Onset: 19 mo after HSCT; Clinic: headaches, tremor, memory loss; MRI: areas of confluent and asymmetrical hyperintensity on T2WI and FLAIR in the bilateral cerebral white matter; CSF: pleocytosis, protein elevation, and low glucose Gadolinium-enhanced; MRI: multiple punctate and curvilinear lesions	Perivascular infiltration of CD3+ CD8+ lympho-myonuclear cells, non-caseating granulomas, loss of myelin and axon preservation	Corticosteroids and tacrolimus	CR
2018 (30)	60	m	AML	Acute GvHD	Onset: 7 d after HSCT; Clinic: impaired consciousness, psychomotor agitation; CSF: elevated protein; MRI: multiple hyperintense lesions on T2 and fluid-attenuated inversion recovery of the deep white matter in frontal as well on the splenium of the corpus callous	Autopsy after CR showed no lymphocytic infiltration in the central nervous system.	Intrathecal methylprednisolone 40mg/w	CR Deceased 5 mo after neurological symptoms of invasive bronchopulmonary aspergillosis
2018 (31)	58	m	ALL	Chronic GvHD	Onset: 1 y after HSCT; Clinic: bradypsychia, cognitive impairment, dyspraxia, ataxia, a pyramidal syndrome, autonomic dysfunction; MRI: infra- and supratentorial leukoencephalopathy, stable in comparison with 1 year before; EEG: diffuse moderate slowing of the dominant rhythm without paroxysmal activity; CSF: elevated protein, oligoclonal bands, elevated IgG; Serum analyses: Caspr2+; PET-CT: diffuse cortical and subcortical hypometabolism	No	MP 1g/d(5d), Cy 500 mg/m^2^/3w, rituximab 375 mg/m^2^/w(4 administrations)	CR Alive 13 mo after CNS symptoms
2018 (32)	59	f	CLL	Acute and chronic GvHD	Onset: 33 mo after HSCT; Clinic: right upper extremity weakness, dysarthria; MRI: left frontal cortex infarction; MRA: severe stenosis of the left MCA; CSF: mild pleocytosis	Inflammatory cell infiltrations of perivascular areas	Surgical revascularization	Clinical and MR stability Alive 6 mo after CNS symptoms
2019 (33)	37	f	MDS	Chronic GvHD	Onset: 2 y after HSCT; Clinic: generalized tonic-clonic type seizures, headache, blurred vision, symmetrical lower leg weakness, increased tendon reflex, hypoesthesia; MRI: multifocal ring enhancement lesions consistent with demyelinating features; multifocal nodular leptomeningeal enhancement and nodular intramedullary enhancing lesions along the spinal cord; CSF: elevated IgG	A loss of myelin fibers, perivascular T-cell infiltration (CD3+), macrophage infiltration associated with reactive gliosis	MP 1g/d(7d), plasmapheresis	CR Alive 1 y after CNS symptoms
2019 (34)	68	m	MDS	No	Onset: 742 d after HSCT; Clinic: right-sided pyramidal distribution of weakness and a stimulus sensitive, myoclonus, increased tendon reflexes; MRI: multiple scattered T2 FLAIR hyperintense, cortical and subcortical lesions in both cerebral hemispheres; CSF: elevated protein EEG: diffuse slow wave activity	Perivascular T-cell infiltration (CD3+), microglial proliferation	MP 1g/d(5d), plasmapheresis	PR then secondary aggravation Deceased 195 d after CNS symptoms

*ALL, acute lymphoblastic leukemia; AML, acute myelogenous leukemia; CLL, chronic lymphocytic leukemia; CML, chronic myelogenous leukemia; CNS, central nervous system; CR, complete response; CSF, cerebrospinal fluid; EEG, electroencephalogram; GvHD, graft versus host disease; HLH, hemophagocytic lymphohistiocytosis; HSCT, hematopoietic stem cell transplantation; MDS, myelodysplastic syndrome; MPN, myeloproliferative neoplasms; MRI, magnetic resonance imaging; NA, not available; PR, partial response*.

**Table 2:** Cerebrospinal fluid test results.

**Cerebrospinal fluid test results**	**Number percentage** **(*n* = 40)**
Protein elevates	23 57.5%
Pleocytosis	13 32.5%
Positive oligoclonal bands	7 17.5
IgG increase	5 12.5%
Low glucose	1 2.5%
Normal	11 27.5%
Not available	5 12.5%

The histological changes of lymphocytic vasculitis are common in skin GvHD, and the specific manifestation is the infiltration of lymph plasma cells around blood vessels and appendages (36). We found that lymphocytic vasculitis has a prominent position in the existing CNS-GvHD histological data. Interestingly, the imaging manifestations of primary and secondary central nervous system vasculitis found in the past are mostly multiple cerebral infarctions, multiple white matter lesions and multiple cerebral hemorrhages, which are also consistent with many current imaging manifestations of CNS-GvHD (37, 38). At present, there is insufficient evidence for whether there is a connection between CNS-GvHD and central nervous system vasculitis, but we will introduce the results of animal model studies in the following section.

Based on the treatment of the above cases, steroid-based immunosuppressive therapy is currently the main first-line therapy for acute and chronic GvHD. These therapies are limited by drug toxicity, non-specific immunosuppression, and long-term treatment requirements. The development of new therapies to improve efficacy and reduce steroid complications is still urgent.

An in-depth understanding of the regulatory cells and molecular pathways involved in the immune response creates opportunities for us to use these cell subsets to prevent GvHD. At present, a variety of cell subsets with immunomodulatory effects, including Treg cells and mesenchymal stem cells (MSC), are being regarded as promising new treatment approaches for GvHD (39). A meta-analysis showed that compared with conventional treatment, MSC infusion showed a significant improvement in the complete remission of chronic GvHD and overall survival, but there was no substantial improvement in the incidence and rate of acute GvHD (40). And, it shows a positive effect for patients who already have acute GVHD (40).

### Possible Mechanism of GvHD

Reviewing these cases, we found that the most common clinical manifestations of patients with CNS graft-versus-host disease were cognitive dysfunction, cranial neuropathy, and seizure. The histology of these cases showed that high frequency of infiltration around the blood vessels (7, 8, 10, 17–19, 22, 27, 29, 32–34), followed by activation or proliferation of microglia, demyelination, infiltration of CD3+/CD8+ T cells (18, 19, 22, 27, 34). In two of them, perivascular infiltrating T cells were confirmed to be donor sources. Animal studies have explored the possible pathological mechanisms.

#### MHC Molecular Expression in CNS

MHC class I molecules on target cell provide specific fragment of foreign for cytotoxic T cells, while MHC class II molecules located on antigen rendering cells are involved in activating helper T cells (41, 42). T lymphocytes and the cellular expression of major histocompatibility complex gene products is practically undetectable in the central nervous system of healthy animals (43). In a parental lymphocyte induced GvHD F1 hybrid rat model, immunohistochemical examination of the central nervous system showed that host class I and II MHC-encoded cell surface molecules were widely expressed in parenchyma and blood vessels. Moreover, scattered T lymphocytes were occasionally found in the central nervous system of these animals (43). Another GvHD F1 hybrid rat model induced by parental splenocytes confirmed that almost all microglia in the rat brain expressed MHC class II molecules 10 days after injection (44).

#### Microglia Activation

Parenchymal lymphocyte inflammation, microglia activation and mild cerebrovascular inflammation-like changes were observed in allogeneic transplanted animals (45). A recent study provided a new possibility to explain the role of microglia activation in CNS-GvHD (46). It pointed out that the expression of MHC class II molecules and CD80 and the productions of TNF were increased on the microglia of mice with GVHD (46). However, in glial cells induced GvHD mice with TNF genetic deletion in, cortical and meningeal infiltration of CD3^+^ T cells was significantly reduced (46). RNA-seq analysis of microglia cells in GvHD models found enhanced proinflammatory MAPK/NF-κB/TAK1 signal in microglia cells and activated various signal cascades in immune cells (46). MHC-unmatched bone marrow or splenocytes induced BALA/c mouse GvHD model revealed significant increased levels of IFN-γ, TNF-α, and IL-6 mRNA in the brain tissue (47). Therapeutic TAK1 inhibition can reduce microglial inflammation during CNS-GvHD (46).

#### T Lymphocyte Infiltration

To establish a mouse model of acute GvHD, allogeneic T cells removed bone marrow was used in allogeneic HCT, while syngeneic were used as the control group (48). It was observed that in the GvHD model, CD3^+^ and CD4^+^ T cells were always found in the cortex, hippocampus, midbrain (thalamus, hypothalamus, basal ganglia), cerebellum, medulla oblongata, choroid plexus (48). CD3^+^ T lymphocytes were also detected in meninges, ependyma, spinal cord neuropil, and sciatic nerve (48). However, absolute numbers showed that the parenchymal infiltration of all these parts is equal, which is equivalent to infection, ischemia and the model of spontaneous inflammatory disease is consistent (48). A study of GvHD rhesus macaques models proved that brain is the target of GvHD in the rhesus macaques transplant model, and CNS GvHD is mainly mediated by CD8^+^ T cells expressing integrins (49). Additionally, CD163^+^ cells (macrophages/monocytes) have also been observed centrally infiltrated in GvHD, suggesting that infiltration of antigen-presenting cells may also be a pathological feature of CNS GvHD (49). They observed two GvHD prevention programs (tacrolimus/methotrexate or rapamycin monotherapy), both of which significantly reduced GvHD-related CD8^+^ infiltration, but failed to completely inhibit the proliferation of CD8^+^ T lymphocytes (49). In the allogeneic HCT mice model, the pathological manifestations of chronic CNS-GvHD was described as diffuse infiltration of the parenchyma and pia mater of CD45+ cells, perivascular inflammation, and microglia activation (45).

#### Increased Inflammatory Cytokines

A study published in 2013 also pointed out that the recipients of allogeneic HCT had significantly lower brain weight than the syngeneic HCT (syngeneic hematopoietic stem cell transplantation) control group, and significant neuronal and glial cell apoptosis (48). This reminds us of the imaging features of brain atrophy in some patients in clinical case reports. After evaluation, it was confirmed that the spatial learning and memory abilities of these GvHD model mice were significantly impaired (48). In experiments with rhesus macaques as experimental subjects, the animals showed obvious significant lassitude, and acute behavioral depression (49). In animal models, IL-6 is related to mood and cognitive dysfunction, and IL-6 receptors are expressed on microglia and astrocytes in the brain (50, 51). For the performance of abnormal animal behavior, researchers had conducted a more in-depth study with host interleukin 6 production as the entry point. The forced swimming test was used to evaluate animal behavior. It was observed that the struggle behavior of GVHD animals in the test increased significantly, which is a dysfunctional response (47). Mice given anti-IL-6R antibodies significantly reduced this effect (47). Blocking the IL-6 pathway can significantly reduce the accumulation of donor T cells, the expression of inflammatory cytokines and the proliferation of host microglia, but it cannot reverse all metabolic abnormalities during GvHD (47). This study explains why drugs that inhibit the peripheral IL-6 signaling pathway, such as tocilizumab, may not completely eliminate the neuroinflammation that occurs in GVHD patients (47).

#### Other Findings

Another study used parental strain's lymph node cells injecting into genetically tolerated F1 hybrid newborn rats to induce GvHD showed that the total cytoplasmic RNA of the cerebellum and the biological activity of each unit of RNA in the experimental group was found decreased (52). The early gene product c-Fos is a reliable sign of neuron activation, and peripheral messengers generated during graft versus host reaction can directly or indirectly trigger the initial activation of neurons in a specific region of the brain (53). The researchers observed a strong c-Fos immune response in the piriform cortex and several other olfactory-related areas (IGr, TT, AO, Ent), occipital visual cortex, and prefrontal cortex (53). The relationship between these changes and the clinical manifestations of GvHD patients had not been confirmed.

### Case Discussion

Compared with the previous cases, our case is a particular one. The patient's clinical characteristics met the diagnostic criteria for chronic CNS-GvHD, but the up to 3 years delayed diffuse white matter lesions shown in MRI were not common in previous cases. This particular imaging change is different from encephalitis and does not have the characteristic of bilateral symmetrical diffuse lesions, which is closer to the change of poisoning or metabolic encephalopathy. Significantly high intracranial pressure and swelling of brain have not been reported in previous cases. In addition, the patient also exhibited chronic GvHD of the skin that coincided with symptoms of the central nervous system, suggesting the CNS manifestations might be explained by the same reason. After starting empirical immunosuppressive therapy, MRI lesions and clinical symptoms of both CNS and skin were completely relieved, which also confirmed that GvHD might be etiologic factors of both skin and CNS damage. Unfortunately, the patient's brain tissue biopsy could not be performed, while according to previous case reports and basic research, we speculate that the cause of the patient may be related to the perivascular inflammation caused by GvHD and the destruction of the blood-brain barrier. If chronic GvHD causes a large number of inflammatory factors and inflammatory cells released into the blood, it may spread into the brain tissue through the destroyed blood-brain barrier, causing white matter degeneration and brain swelling, which in turn leads to the occurrence of high intracranial pressure. With the start of immunosuppressive therapy, the patient's symptoms improved rapidly, and the cerebrospinal fluid pressure dropped to normal level.

The patient's nervous system has been relieved in imaging and symptoms. The treatment produced a very rapid treatment response and was well tolerated by the patient. With subsequent treatment, there should be an optimistic prognosis in terms of CNS-GvHD. However, she still faces the risk of disease recurrence, complications, infection and drug side effects in the future.

## Data Availability Statement

The original contributions presented in the study are included in the article/Supplementary Material, further inquiries can be directed to the corresponding authors.

## Ethics Statement

Written informed consent was obtained from the individual(s) for the publication of any potentially identifiable images or data included in this article.

## Author Contributions

ML performed case information collection, literature review, literature information statistics, and drafted the manuscript. YZhan contributed to case information collection and literature information statistics. YG, ZZ, and HDon contributed to literature review and manuscript preparation. YZhao and HDen performed manuscript review and final version approval. All authors contributed to the article and approved the submitted version.

## Conflict of Interest

The authors declare that the research was conducted in the absence of any commercial or financial relationships that could be construed as a potential conflict of interest.
