# Supplementary figures and images for: A Case Report of Central Nervous System Graft-Versus-Host Disease and Literature Review
Source: Front Neurol. 2021 Mar 10;12:621392. doi: 10.3389/fneur.2021.621392 (PMC7987907; doi:10.3389/fneur.2021.621392)

# Supplementary Chart 1

## Proportion of Different Primary Diseases

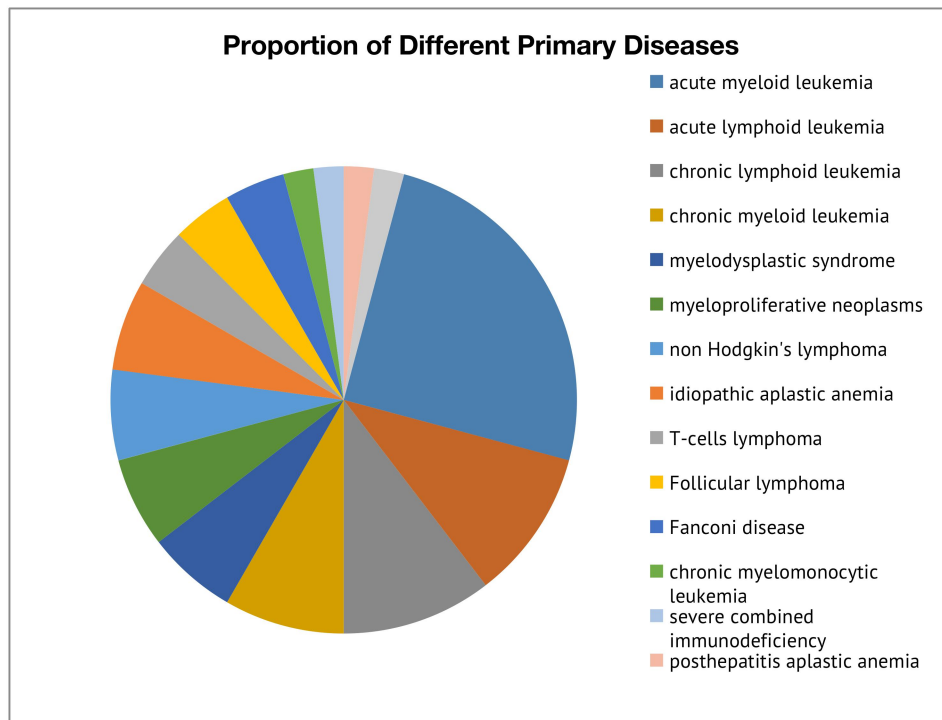

Supplement: Supplementary file 1 [file Data_Sheet_1.pdf]

## Supplementary hart 2

### Clinical Manifestations of Central Nervous System

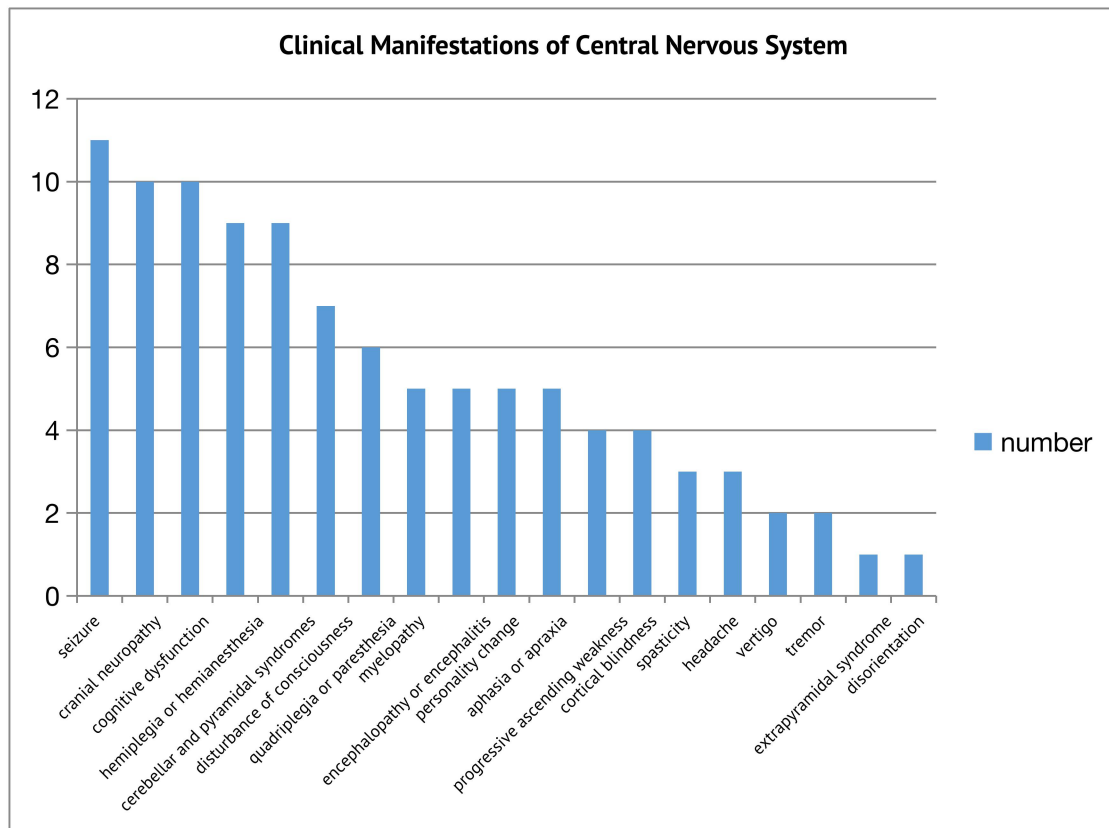

Supplement: Supplementary file 2 [file Data_Sheet_2.pdf]
