# Supplementary material for: A Case Report of Central Nervous System Graft-Versus-Host Disease and Literature Review
Source: Front Neurol. 2021 Mar 10;12:621392. doi: 10.3389/fneur.2021.621392 (PMC7987907; doi:10.3389/fneur.2021.621392)
Supplement: Supplementary file 3 [file Table_1.DOCX]

| **Supplementary Table 1**  **Imaging Characteristics** | | |
| --- | --- | --- |
| Imaging characteristics | | Number  N=46 |
| White matter lesions | | 30 |
|  | unilateral or bilateral asymmetric white matter lesions | 15 |
|  | multiple sclerosis | 7 |
|  | ADEM | 6 |
|  | diffuse white matter lesions | 1 |
|  | corpus callosum lesion | 1 |
| Hemorrhagic or ischemic (4 with white matter lesions) | | 9 |
| Brain atrophy (1 with white matter lesions) | | 4 |
| Not specific |  | 4 |
| Normal |  | 3 |
| Not available |  | 1 |
