# Supplementary material for: A Case Report of Central Nervous System Graft-Versus-Host Disease and Literature Review
Source: Front Neurol. 2021 Mar 10;12:621392. doi: 10.3389/fneur.2021.621392 (PMC7987907; doi:10.3389/fneur.2021.621392)
Supplement: Supplementary file 4 [file Table_2.DOCX]

| **Supplementary Table 2**  **Treatment Methods** |  |  |
| --- | --- | --- |
| Treatment methods |  | Number  N=46 |
| Surgical treatment |  | 1 |
| Only corticosteroids |  | 13 |
| Only other immunosuppressive therapy | tacrolimus | 1 |
|  | IV Ig | 1 |
|  | cyclosporin A | 1 |
| Corticosteroids+othrer immunosuppressive therapy | cyclophosphamide | 8 |
| (some had 2 immunosuppressive therapy) | plasma exchange | 5 |
|  | IV Ig | 5 |
|  | cyclosporin | 3 |
|  | mycophenolate mofetil | 2 |
|  | rituximab | 1 |
|  | fingolimod | 1 |
|  | methotrexate | 1 |
|  | etoposide | 1 |
|  | tacrolimus | 1 |
| Untreated |  | 5 |
