# Supplementary material for: A Case Report of Central Nervous System Graft-Versus-Host Disease and Literature Review
Source: Front Neurol. 2021 Mar 10;12:621392. doi: 10.3389/fneur.2021.621392 (PMC7987907; doi:10.3389/fneur.2021.621392)
Supplement: Supplementary file 5 [file Table_3.DOCX]

| **Supplementary Table 3**  **Prognosis Condition and Follow Up** | | | |
| --- | --- | --- | --- |
| Treatment methods | Pathogenetic condition | Number  N=46 |  |
| Surgical treatment (n=1) | Stable | 1 |  |
| Immunosuppressive therapy (n=40) | CR | 15 |  |
|  | PR | 16 |  |
|  | Progress | 5 |  |
|  | Stable | 2 |  |
|  | Transient improvement | 2 |  |
| Untreated (n=5) | PR | 1 |  |
|  | Progress | 1 |  |
|  | NA | 3 |  |

CR=complete response, NA=not available, PR=partial response.
